# Understanding the patient journey to diagnosis of lung cancer

**DOI:** 10.1186/s12885-021-08067-1

**Published:** 2021-04-14

**Authors:** Yichen Zhang, Michael J. Simoff, David Ost, Oliver J. Wagner, James Lavin, Beth Nauman, Mei-Chin Hsieh, Xiao-Cheng Wu, Brian Pettiford, Lizheng Shi

**Affiliations:** 1 Department of Health Policy and Management, School of Public Health and Tropical Medicine, Tulane University, 1440 Canal Street, Suite 1900, New Orleans, LA 70112 USA; 2 Bronchoscopy and Interventional Pulmonology, Lung Cancer Screening Program, Pulmonary & Critical Care Medicine, Henry Ford Hospital, Wayne State University School of Medicine, 2799 West Grand Boulevard, Detroit, MI 48202 USA; 3 Department of Pulmonary Medicine, University of Texas MD Anderson Cancer Center, Houston, TX 77030 USA; 4 Intuitive, 1020 Kifer Road, Sunnyvale, CA 94086 USA; 5 Louisiana Public Health Institute, 1515 Poydras Street #1200, New Orleans, LA 70112 USA; 6 Louisiana State University Health Science Center, 433 Bolivar St, New Orleans, LA 70112 USA; 7 Ochsner Health System, 1514 Jefferson Highway, Jefferson, LA 70121 USA

**Keywords:** Lung cancer, Clinical pathway, Bronchoscopy, Pulmonary nodule, Biopsy frequency, Staging

## Abstract

**Objective:**

This research describes the clinical pathway and characteristics of two cohorts of patients. The first cohort consists of patients with a confirmed diagnosis of lung cancer while the second consists of patients with a solitary pulmonary nodule (SPN) and no evidence of lung cancer. Linked data from an electronic medical record and the Louisiana Tumor Registry were used in this investigation.

**Materials and methods:**

REACHnet is one of 9 clinical research networks (CRNs) in PCORnet®, the National Patient-Centered Clinical Research Network and includes electronic health records for over 8 million patients from multiple partner health systems. Data from Ochsner Health System and Tulane Medical Center were linked to Louisiana Tumor Registry (LTR), a statewide population-based cancer registry, for analysis of patient’s clinical pathways between July 2013 and 2017. Patient characteristics and health services utilization rates by cancer stage were reported as frequency distributions. The Kaplan-Meier product limit method was used to estimate the time from index date to diagnosis by stage in lung cancer cohort.

**Results:**

A total of 30,559 potentially eligible patients were identified and 2929 (9.58%) had primary lung cancer. Of these, 1496 (51.1%) were documented in LTR and their clinical pathway to diagnosis was further studied. Time to diagnosis varied significantly by cancer stage. A total of 24,140 patients with an SPN were identified in REACHnet and 15,978 (66.6%) had documented follow up care for 1 year. 1612 (10%) had no evidence of any work up for their SPN. The remaining 14,366 had some evidence of follow up, primarily office visits and additional chest imaging.

**Conclusion:**

In both cohorts multiple biopsies were evident in the clinical pathway. Despite clinical workup, 70% of patients in the lung cancer cohort had stage III or IV disease. In the SPN cohort, only 66% were identified as receiving a diagnostic work-up.

**Supplementary Information:**

The online version contains supplementary material available at 10.1186/s12885-021-08067-1.

## Background

Lung cancer is the leading cause of cancer-related deaths in the United States, causing more deaths than colorectal, breast, and prostate cancers combined [1]. An estimated 135,720 Americans are expected to die of lung cancer in 2020, accounting for about 22% of all cancer deaths in the United States [1]. The 5-year relative survival rate for patients with lung cancer is poor and varies considerably from 59% for patients with localized disease, 31.7% for patients with regional disease, and 5.8% for people with distant disease. The relative 5-year survival for those with stage unknown is 8.3% [2]. Trends in relative survival have improved slightly from 12.5% in 1975–1979 to 19.9%% in 2012 attributable to changes in smoking patterns and therapeutic improvements [1]. Routine screening for lung cancer has not yet been broadly adopted despite evidence that screening high risk patients (i.e., 30 pack years of cigarette smoking) with the use of low-dose CT reduces mortality from lung cancer [3, 4]. Engaging primary care clinicians and garnering support from payors to ensure routine lung cancer screening adoption will have a significant impact on overall patient survival [5]. Currently, most lung cancer (84%) is diagnosed with regional or distant extension of the disease, limiting treatment options and reducing relative survival [2].

Patients with lung cancer may follow a variety of routes within the health-care system. Traditionally, consultation and management have been achieved with referrals to specialists occurring in a sequential fashion. Symptoms prompt a physician visit, followed by a referral to a specialist culminating in a diagnosis and treatment. This process may be slow, and delays are common. Asymptomatic patients with a solitary pulmonary nodule may suffer even longer delays as the guidelines recommend watchful waiting rather than more aggressive diagnostic procedures especially for those nodules < 8 mm [6]. Previous studies of diagnostic delay in lung cancer (i.e., abnormal chest imaging to diagnosis) have shown a median delay between 25 and 53 days [7–9] but delays have been shown to be skewed with some patients having much longer delays [9, 10]. Currently, lung cancer diagnosis and staging have increased in complexity due to an expanding number of options such as PET imaging, endobronchial ultrasound, as well as navigational and robotic bronchoscopy. These tools allow for more extensive tissue sampling and accurate diagnosis and staging. Patients with early-stage disease are often eligible for potentially curative therapy with minimally invasive surgery and advanced radiotherapy techniques. For patients with more advanced disease, molecular profiling for tumor mutations may guide the use of targeted therapies such as ALK, BRAF, ROS1, RET, NTRK, MET (with EGFR inhibitors) and anti PD-1/PD-L1 immune checkpoint inhibitors [11]. Such molecular analysis may require additional tissue sampling or sample analysis at a remote specialty lab. These factors, among others in the patient journey, indicate there is no singular pathway to a diagnosis of lung cancer and add to the variability of timely diagnosis [12–15].

We conducted a retrospective cohort study using data from the electronic health records of Tulane Medical Center and Ochsner Health System linked to the Louisiana Tumor Registry to document the patient pathway to lung cancer diagnosis and report the pattern of procedures and physician visits. For patients with lung nodules ultimately diagnosed as malignant we report the median time to diagnosis. For patients with lung nodules diagnosed as benign, we report on diagnostic lung procedures performed during the subsequent 1-year from nodule identification.

## Methods

This is a retrospective cohort study, using linked data information systems, designed to document the patient pathway from (a) identification of a pulmonary nodule to lung cancer diagnosis, and (b) identification of a pulmonary nodule to 1-year follow-up among those with no lung cancer diagnosis.

### Datasets

*The Louisiana Tumor Registry (LTR)* is a population-based cancer registry that is authorized by law to collect data on cancer diagnosis, treatment, and survival from all healthcare facilities and providers. The LTR is a participant of the NCI’s Surveillance, Epidemiology, and End Result Program and the CDC’s National Program of Cancer Registries. The LTR’s data have met the data completeness, timeliness, and quality standards for both SEER and NPCR programs and the North American Association of Central Cancer Registries [16].

*The Research Action for Health Network (REACHnet)* is a partnership of health systems, academic centers, and public health organizations that constitute an innovative data network for conducting multi-site research with enhanced efficiency in real-world healthcare delivery systems. With national and local collaborators, REACHnet implements research that addresses healthcare questions of critical importance to patients and clinicians and contributes to the evidence base that will inform more effective healthcare decision-making and improve population health. The network includes clinical records for more than 8 million patients across multiple partner health systems located in Louisiana, Texas, and California. For the current study, data were obtained through REACHnet (https://reachnet.org/) for qualifying patients receiving care at Ochsner Health System and Tulane Medical Center in Louisiana.

### Data linkage

Data linkage between these two information systems was performed using a Global Patient Identification (GPID), provided by REACHnet. A software application, Distributed Common Identity for the Integration of Regional Health Data (DCIFIRHD), was used to perform secure, cross-site aggregation and linkage of patient records between LTR and REACHnet. Patient identification information (e.g., first name, last name, date of birth, SSN, gender), was hashed in various combinations with a site-specific password and a shared seed to create up to 17 hashes for each patient. These Seeded Hash Identifiers (HashIDs) were sent to an honest broker site, REACHnet, where the hashed output was merged using a deterministic algorithm that applied sets of rules to the Seeded HashIDs of common patient identifiers to identify matches between records. Two records from REACHnet and LTR with the same Seeded HashID were considered a “match” (i.e., be the same patient) if they had the same value for certain Seeded HashIDs. Unique study IDs were then used to replace the matched HashIDs [16]. A primary lung cancer diagnosis in REACHnet was identified using ICD-9-CM (International Classification of Diseases, Ninth Revision, Clinical Modification) and ICD-10-CM (International Classification of Diseases, Tenth Revision, Clinical Modification) diagnosis codes. Patients with primary lung cancer diagnosis in LTR were identified using ICD-O-3 (International Classification of Diseases for Oncology, Third Revision) codes C340-C349.

### Study population

**Fig. 1:**
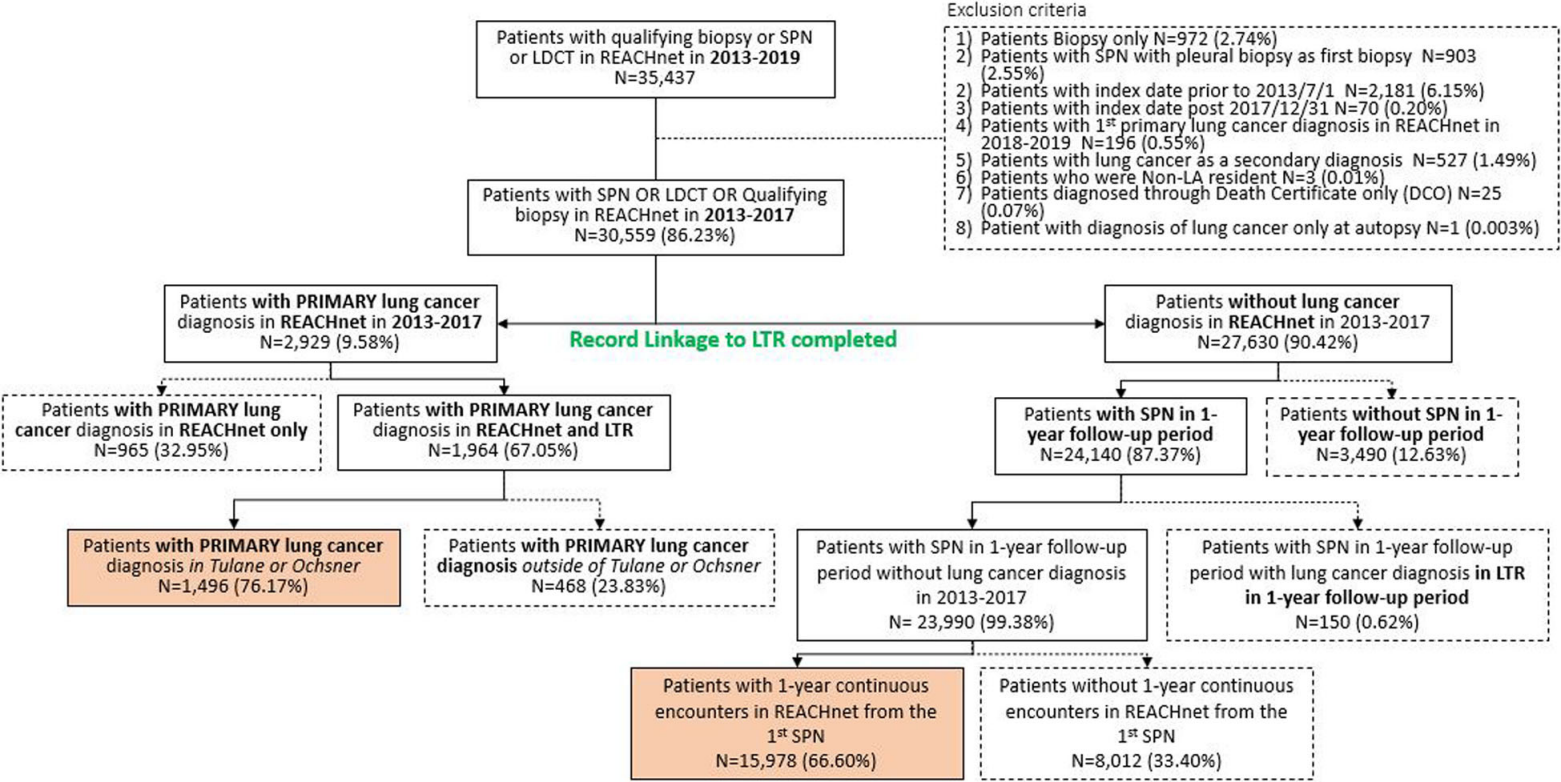
Sample selection flow chart

Our goal was to identify all patients in Tulane and Ochsner healthcare systems who had either a malignant or benign lung nodule between 2013 and 2019. Figure 1 shows the patient selection process and the resulting study populations. Patients with (a) a qualifying biopsy defined by Current Procedural Terminology (CPT) codes or (b) a diagnosis of Suspicious Pulmonary Nodule (SPN) or (c) Low-Dose Computed Tomography (LDCT) defined by ICD-9 or ICD-10 codes in REACHnet between 2013 and 2019 were identified and the first recorded date of SPN or LDCT or chest imaging was defined as the index date (see Appendix 1 for code lists). Patients were excluded from further assessment if (a) they had a Biopsy only *N* = 972 (2.74%), (b) they had a SPN with pleural biopsy as first biopsy *N* = 903 (2.55%), (c) their index date occurred prior to 1/7/2013 *N* = 2181 (6.15%), (d) their index date was after 12/31/2017 *N* = 70 (0.20%) (linkage to LTR was not available after 12/31/17), (e) their diagnosis with primary lung cancer diagnosis in REACHnet occurred in 2018–2019 *N* = 196 (0.55%), (f) lung cancer was a secondary diagnosis *N* = 527 (1.49%), (g) they were not Louisiana residents *N* = 3 (0.01%), (h) they were diagnosed through Death Certificate only (DCO) *N* = 25 (0.07%) and (i) they were diagnosed with lung cancer only at autopsy *N* = 1 (0.003%). A total of 4878 patients met these exclusion criteria and were eliminated from further study. (See Fig. 1) A total of 30,559 patients from REACHnet were linked to the LTR including 2929 patients with a primary lung cancer diagnosis in REACHnet and 27,630 patients without a lung cancer diagnosis in REACHnet between 2013 and 2017. Of the 2929 patients with a lung cancer diagnosis in REACHnet, 1496 had a primary lung cancer diagnosis in both the Tulane or Ochsner health care systems and in the LTR. These 1496 subjects constitute the lung cancer patient population. Records for these 1496 patients were assembled to document patient care from index date to diagnosis date. The diagnosis date from the LTR was used as the study endpoint for the lung cancer cohort and was defined using the earliest date of cytology, or biopsy, or staging biopsy date in the LTR. If none of these dates were available, then the diagnosis date as recorded in the LTR was used.

Of the 27,630 patients without a lung cancer diagnosis in REACHnet between 2013 and 2017, 3490 had no evidence of SPN at baseline (i.e., qualified for study inclusion based on a biopsy or LDCT) or during the 1 year of follow-up. In addition, a total of 150 patients had evidence of lung cancer following data linkage with LTR, presumably having left Tulane or Ochsner healthcare systems to receive care elsewhere. Of the remaining 23,990 patients with SPN at baseline or during the 1-year follow-up, a total of 15,978 had continuous enrollment in REACHnet during the 1-year follow-up period and no evidence of a lung cancer diagnosis. These subjects constitute the SPN patient population. The medical records for these 15,978 patients were assembled to document care from the first recorded SPN (index date) to 1 year from the initial SPN diagnosis date.

### Patient pathway

Utilization of invasive and non-invasive procedures until diagnosis in the lung cancer cohort and during the first year following SPN diagnosis in REACHnet were extracted using ICD-9-CM procedure codes, ICD-10-CM procedure codes, and Current Procedural Terminology (CPT) codes. Procedures under investigation in this study included Evaluation and Monitoring (E&M), Biopsy (i.e., surgical biopsy, CT guided biopsy, and bronchoscopy biopsy), Positron Emission Tomography-Computed Tomography (PET CT), Brain Magnetic Resonance Imaging (MRI), and Bone Scan (Appendix 1).

Biopsy associated complications including pneumothorax, pneumothorax requiring a chest tube, hemorrhage, and air leak were extracted using ICD-9-CM diagnosis codes, ICD-10-CM diagnosis codes, CPT procedure codes, ICD-9-CM procedure codes, and ICD-10-CM procedure codes (Appendix 1).

### Statistical analysis

Descriptive analysis for demographic characteristics was performed. Demographic characteristics for patients with lung cancer were compiled from data from the LTR data, while data for patients with SPN diagnoses without lung cancer were compiled from REACHnet’s electronic health record (EHR) data. Continuous variables were reported in mean and standard deviation (SD). Categorical variables were reported in frequency and percentage distribution. Health services utilization rates were reported in frequency and percentage distribution. Time from index date to diagnosis date by stage was analyzed using the Kaplan-Meier product limit method. All analyses were performed in SAS 9.4 (SAS Institute, Cary, North Carolina, USA).

## Results

Table 1 shows selected demographics by patient cohort. While direct comparisons between cohorts are problematic given the differences in selection criteria and follow-up, patients in the lung cancer cohort were older with a preponderance of men relative to patients in the SPN cohort. As would be expected, patients in the lung cancer cohort had, on average, more invasive procedures relative to the SPN cohort however patients in the SPN cohort had more non-invasive procedures relative to the lung cancer cohort attributable, in part, to the longer observation period for patients in the SPN cohort.

**Table 1:** Selected demographic characteristics by cohort

	Patients with w/EMR at Tulane and Ochsner and have confirmed diagnosis in LA tumor registry (***N*** = 1496)	Patients without primary lung cancer with SPN and 1-year follow up (***N*** = 15,978)
**Age, mean (SD)**	68.41 (10.03)	61.74 (15.19)
**Age group, N (%)**		
18–34 years old	4 (0.27)	958 (6.00)
35–44 years old	14 (0.94)	1186 (7.42)
45–54 years old	100 (6.68)	2351 (14.71)
55–64 years old	382 (25.53)	4138 (25.90)
≥ 65 years old	996 (66.58)	7345 (45.97)
**Gender, N (%)**		
Male	843 (56.35)	7139 (44.68)
Female	651 (43.52)	8839 (55.32)
**Race, N (%)**		
White	976 (65.24)	10,461 (65.47)
Black	504 (33.69)	5225 (32.70)
^a^Other	16 (1.07)	292 (1.83)
**Region, N (%)**		
Outside New Orleans	258 (17.25)	n/a
New Orleans Area	1237 (82.69)	
**Health insurance type at diagnosis, N (%)**		
Medicare	1010 (67.51)	n/a
Private Insurance	235 (15.71)	
Medicaid	92 (6.15)	
Insurance not specified	76 (5.08)	
TRICARE/Veterans Affairs	49 (3.28)	
Not insured	24 (1.60)	
Insurance status unknown	10 (0.67)	
**Year of diagnosis, N (%)**		
2013	165 (11.03)	n/a
2014	312 (20.86)	
2015	317 (21.19)	
2016	347 (23.20)	
2017	355 (23.73)	
**AJCC stage, N (%)**		
Stage 0	9 (0.60)	n/a
Stage I	308 (20.59)	
Stage II	108 (7.22)	
Stage III	327 (21.86)	
Stage IV	707 (47.26)	
Unknown, not staged	37 (2.47)	
**Overall:**		
Average number of invasive procedures^b^ from index date to diagnosis date or end of study period^c^, mean (SD)	2.11 (0.75)	1.58 (1.66)
Average number of non-invasive procedures from index date to diagnosis date or end of study period^c^, mean (SD)	5.88 (7.68)	10.47 (9.68)

^a^Asian, American Indian, Aleutian, or Eskimo

^b^biopsies, EBUS and Mediastinoscopy

^c^Observation periods for each population are different. In the Lung cancer population, index date to diagnosis date. In the SPN population, the index date to the end of 1 year follow up period

All imaging (cpt 71,250, 71,260, 71,270, 71,010, 71,020, 74,176, 74,178, GO297, GO296, S8032)

All consultations/office visits (99201–99,205, 99,213–99,215, 99,221–99,223, 99,251–99,254)

Table 2 shows the staging and clinical measurements for lung cancer patients (*n* = 1496) from index date until date of diagnosis stratified by subsequent clinic visit. (Test utilization stratified by stage can be seen in the online appendix). Approximately 15% of lung cancer patients received a diagnosis of lung cancer on their index date while only 8% had biopsy information on their index date. Reasons for the lack of concordance between diagnosis and biopsy information is unknown but likely attributable to patients receiving their index scan and/or biopsy outside of the REACHnet system. The most common clinical measures recorded at this time point were a chest x-ray and an E&M. The presence of other clinical measurements in the medical record was very low. Sixty-nine percent of the patients diagnosed on the index date were Stage 3 or 4. Subsequent visits showed a similar pattern. For example, at visit 1 following the index date, 18% received a diagnosis of lung cancer and 76.6% of these patients were stage 3 or 4. The clinical measurements at visit 1 following the index date showed a decrease in the frequency of chest imaging and increases in the frequency of E&M and biopsy. The frequency of EBUS, PET CT, Brain MRI, and bone scan also increased although the frequency of these measurements was still low (0.6–3.0%). Approximately 38% of all lung cancer patients (*N* = 564) received their diagnosis on or after the fourth visit. Sixty-three percent of these individuals were stage 3 or 4. Chest imaging and biopsy were the most frequently recorded clinical measures followed by E&M, PET CT and EBUS. The use of LDCT, brain MRI, and bone scan was infrequent throughout the patient visitation process.

**Table 2:** Staging and clinical measurements for lung cancer patients from index date by subsequent clinic visit

Visit Characteristics	Patients with confirmed diagnosis of lung cancer, Index Date	Visit one following index visit	Visit two following index visit	Visit three following index visit	Visit 4 + all visits up to end of study period
	*N* = 1496	*N* = 1275	*N* = 1002	*N* = 752	*N* = 564
Days (median (IQR)) between visits		4 (1,11)	5 (1,12)	5 (1,12)	6.5 (1,25)
Cumulative days from index visit (median (IQR))		4 (1,11)	11 (4,25)	20 (8, 51)	48 (16, 268)
LDCT scan	9 (0.60)	3 (0.24)	1 (0.10)	0 (0.00)	2 (0.35)
Chest Imaging	1085 (72.53)	434 (34.04)	331 (33.03)	263 (34.97)	443 (78.55)
E&M	577 (38.57)	675 (52.94)	446 (44.51)	338 (44.95)	379 (67.20)
EBUS/mediastinoscopy	29 (1.94)	42 (3.29)	34 (3.39)	33 (4.39)	106 (18.79)
Biopsy^a^	116 (7.75)	252 (19.76)	250 (24.95)	183 (24.34)	439 (77.84)
PET CT	20 (1.34)	47 (3.69)	69 (6.89)	44 (5.85)	119 (21.10)
Brain MRI	45 (3.01)	33 (2.59)	27 (2.69)	10 (1.33)	44 (7.80)
Bone scan	4 (0.27)	8 (0.63)	6 (0.60)	5 (0.66)	7 (1.24)
SPN diagnosis	1066 (71.26)	786 (61.65)	625 (62.38)	462 (61.44)	515 (91.31)
Lung cancer diagnosis made^b^	221 (14.77)	273 (18.24)	250 (16.71)	188 (12.57)	564 (37.70)
*AJCC Stage at diagnosis*					
*Stage 0*			1 (0.40)		8 (1.42)
*Stage I*	38 (17.19)	47 (17.22)	41 (16.40)	35 (18.62)	147 (26.06)
*Stage II*	20 (9.05)	13 (4.76)	24 (9.60)	14 (7.45)	37 (6.56)
*Stage III*	39 (17.64)	53 (19.41)	56 (22.40)	52 (27.66)	127 (22.52)
*Stage IV*	114 (51.58)	156 (57.14)	124 (49.60)	81 (43.09)	232 (41.13)
*Unknown, not staged*	10 (4.52)	4 (1.47)	4 (1.60)	6 (3.19)	13 (2.30)

^a^all biopsy types from REACHnet database. Excludes EBUS/Mediastinoscopy (counted separately) and biopsy of the pleura

^b^% relative to the total number of cancer patients (*N* = 1496)

**Fig. 2:**
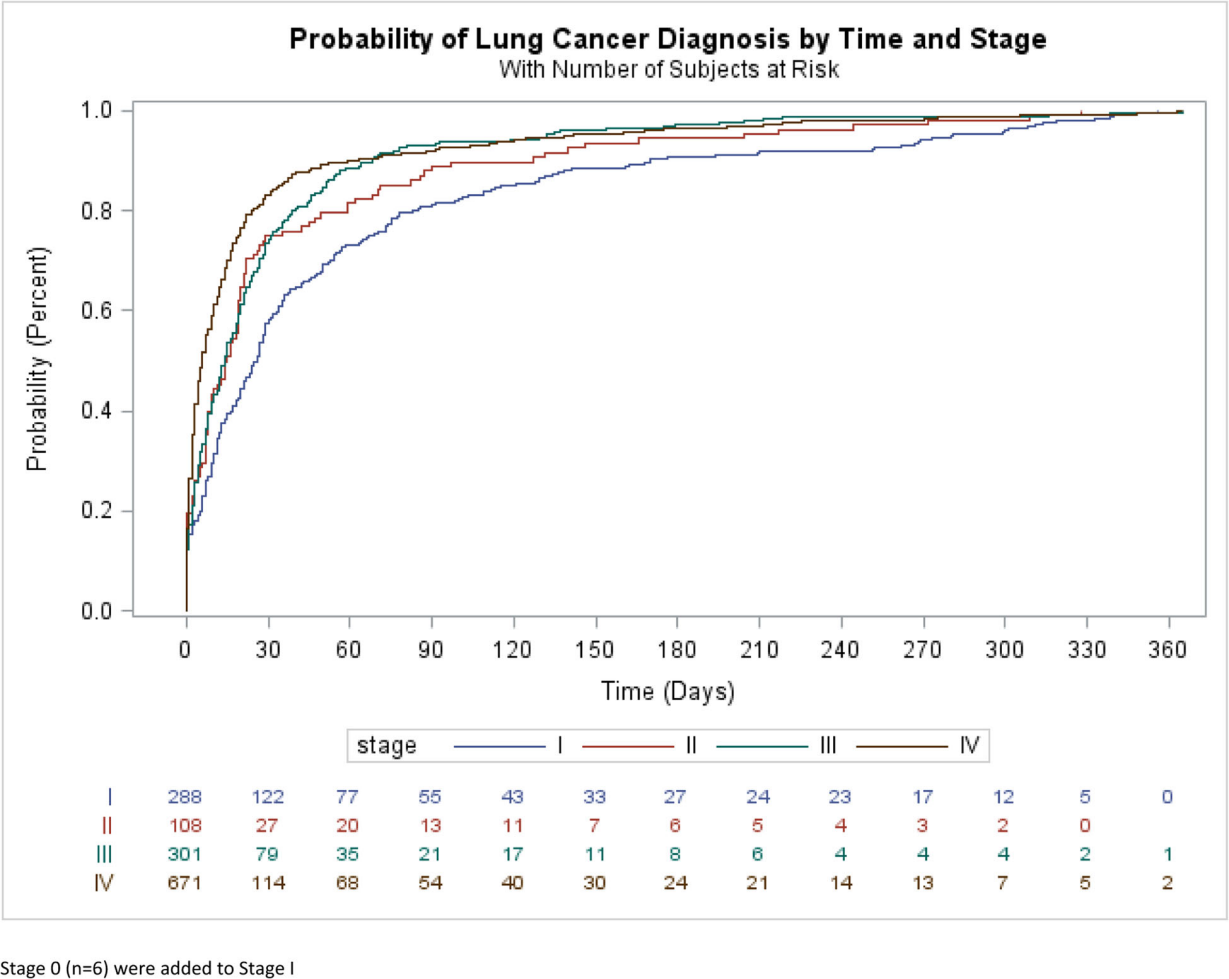
Cumulative probability of diagnosis for lung cancer patients, stratified by AJCC stage (stage I - stage IV)

Figure 2 shows the cumulative probability of diagnosis for lung cancer patients stratified by AJCC stage. Approximately 80% of all stage 3 and 4 lung cancer was diagnosed within a 45-day time period from index date. Eighty percent of stage 2 lung cancers were diagnosed within a period of 60 days and 80% of stage 1 cancers were diagnosed within a period of 90 days. The median time to diagnosis for all lung cancer patients was 13 days with an interquartile range of 2 to 46 days and varied significantly by cancer stage. Approximately 50% of lung cancer patients were diagnosed as stage 4 with a median time to diagnosis of 7 days while stage 3 (22% of the population), stage 2 (7% of the population), and stage 1 (21% of the population) patients’ median times to diagnosis were 16, 16.5 and 33 days respectively. (data not shown). The time from index date to diagnosis was significantly shorter for those with stage 3 or 4 lung cancer compared to those diagnosed at an earlier stage (Log rank test, *p* < 0.0001). Despite the differences in time to diagnosis by lung cancer stage, the tests and procedures on the index date and by visit subsequent to index date are remarkably comparable (see supplemental Table). For example, chest imaging on index date was 67, 68, 73 and 78% for stages 1–4 respectively while biopsy on the index date was 7.6, 13.0, 7.7 and 6.7% respectively.

The pattern of clinical measurements on index date and subsequent visits from index for SPN patients is shown in Table 3. A total of 15,978 patients received a SPN diagnosis and were followed for 1 year to determine subsequent work up characteristics. Ten percent of this population (*n* = 1612) had no evidence of a SPN work-up following the index SPN diagnosis. The remaining 14,366 (89.9%) had some evidence of an SPN workup in their medical record the majority of which was E&M and additional chest imaging. Among this group, the recording of LDCT, EBUS, or bone scan was infrequent (< 1%) while the use of biopsy, PET CT and brain MRI ranged from 3 to 6%. Approximately 65% of this patient cohort was seen 4 or more times during the follow-up period and this group tended to have more procedures and documentation of SPN workup compared to those with 3 or fewer visits.

**Table 3:** Clinical measurements for SPN patients from index date by subsequent clinic visit

Visit Characteristics	Patients with SPN and no diagnosis of Lung Cancer	Visit one following index visit	Visit two following index visit	Visit three following index visit	Visit 4 + all visits up to end of study period
	*N* = 15,978	*N* = 14,366	*N* = 13,009	*N* = 11,715	*N* = 10,476
Days (median (IQR)) between visits		11 (3, 42)	16 (4, 50)	17 (5, 49)	32.5 (19.3, 56.1)
Cumulative days from index visit (median (IQR))		11 (3, 42)	38 (12, 104)	67 (25, 151)	196.4 (148.6, 251.5)
LDCT scan	26 (0.16)	4 (0.03)	3 (0.02)	3 (0.03)	6 (0.06)
Chest Imaging	9874 (61.80)	4868 (33.89)	3623 (27.85)	2931 (25.02)	5717 (54.57)
E&M	6129 (38.36)	9632 (67.05)	9483 (72.90)	8886 (75.85)	9972 (95.19)
EBUS/mediastinoscopy	28 (0.18)	34 (0.24)	23 (0.18)	25 (0.21)	75 (0.72)
Biopsy^a^	187 (1.17)	157 (1.09)	143 (1.10)	96 (0.82)	399 (3.81)
PET CT	99 (0.62)	101 (0.70)	100 (0.77)	62 (0.53)	284 (2.71)
Brain MRI	104 (0.65)	148 (1.03)	107 (0.82)	95 (0.81)	576 (5.50)
Bone scan	7 (0.04)	12 (0.08)	21 (0.16)	15 (0.13)	77 (0.74)
SPN diagnosis	15,978 (100.00)	3597 (25.04)	2432 (18.69)	1818 (15.52)	3558 (33.96)
Loss to follow up, N (%)^b^		1612 (10.09)	1357 (8.49)	1294 (8.10)	1239 (7.75)

^a^biopsies do not include EBUS/mediastinoscopy

^b^% relative to the total number of cancer patients (N = 15,978)

Table 4 shows the distribution of biopsies by type, frequency and cohort (lung cancer, SPN only). A total of 1167 (79.4%) lung cancer patients had biopsy information in their EHR, 48.3% of which were bronchoscopic, 42.8% CT guided and 8.9% surgical. A total of 25.7% had multiple biopsies and the average number of biopsies was 1.25 per patient. A total of 932 (5.83%) of SPN patients had biopsy information, either from their index visit or during follow-up in their EHR, 69.1% of which were bronchoscopic, 19.3% CT guided and 11.6% surgical. A total of 35.5% of these patients had multiple biopsies and the average number of biopsies was approximately 1.36 per patient.

**Table 4:** Patients with record of Biopsy by type^a^

	CT guided biopsy		Bronchoscopy		Surgical biopsy		Total procedures		Unique # of patients	
	lung cancer DX	SPN only	lung cancer DX	SPN only	lung cancer DX	SPN only	lung cancer DX	SPN only	lung cancer DX	SPN only
Total number of procedures, N (%)	628 (42.75)	271 (19.32)	710 (48.33)	970 (69.14)	131 (8.92)	162 (11.55)	1469	1403	1167 (100.00)	932 (100.00)
1st biopsy	500 (42.84)	231 (24.79)	637 (54.58)	618 (66.31)	30 (2.57)	83 (8.91)	1167	932	1167 (100.00)	932 (100.00)
2nd biopsy	110 (42.97)	33 (16.18)	65 (25.39)	114 (55.88)	81 (31.64)	57 (27.94)	256	204	256 (21.94)	204 (21.89)
3rd biopsy	15 (39.47)	7 (8.64)	7 (18.42)	59 (72.84)	16 (42.11)	15 (18.52)	38	81	38 (3.26)	81 (8.69)
> 4 or more biopsy	3 (37.50)	0 (0.00)	1 (12.50)	179 (96.24)	4 (50.00)	7 (3.76)	8	186	6 (0.51)	46 (4.94)

^a^from REACHnet database. Bronchoscopy includes EBUS. Surgical includes mediastinoscopy. Pleural biopsy excluded

The overall complication rates following biopsy procedures was low in both cohorts; 3.2% in the lung cancer cohort and 2.68% in the SPN cohort. (Appendix 2) Pneumothorax following CT guided biopsy was the most common complication reported in 4.3 and 5.2% of lung cancer and SPN patients respectively.

## Discussion

Numerous studies have documented the timeliness of diagnosis and treatment of lung cancer patients, the majority of which come from European Union member countries. In a systematic review of the literature, Olsson et al. identified 49 studies in which at least one time-interval in lung cancer care was reported [17]. Median times to diagnosis ranged from 8 to 60 days dependent upon the starting point in the patient journey; some studies began at the identification of symptoms while others started at the identification of a SPN on x-ray or CT. The US studies included in this systematic review were three, small, single center VA hospital chart reviews that reported a median time from CT to diagnosis of 45 days [18] and median times from “consultation” to surgery of 82 days and 104 days [19, 20]. More recently, Miaga et al. conducted a chart review of 265 veterans who underwent cancer resection from 2005 to 2015 to assess time intervals between nodule identification, diagnosis, and surgical resection; changes in nodule radiographic size over time; final pathologic staging; and reasons for delays in care [21]. They reported a median time from nodule identification to resection of 98 days with an interquartile range of 66–139 days. Yorio and colleagues identified 482 lung cancer patients treated at three hospitals associated with the University of Texas (UT) Southwestern Medical Center and reported a median of 16 days from image to diagnosis with an interquartile range of 6–43 days [22]. Nadpara et al. used National Cancer Institute’s (NCI) Surveillance, Epidemiology, and End Results (SEER)-Medicare linked data files from years 2002–2007 and reported a median of 187 days with an interquartile range of 36–308 days from first recorded symptoms to diagnosis [23]. The difficulty in using first recorded symptoms as the starting point in a patient journey is their lack of specificity. Cough, shortness of breath and chest pain were the most frequently reported symptoms prior to diagnosis in a Canadian study of lung cancer patients; symptoms common to a variety of disease states from asthma to cardiac disease [24]. Our study focused on the time period from first identified SPN, CT image or qualifying biopsy to diagnosis and found a median of 13 days with an interquartile range of 2 to 46 days. However, we found that there are significant differences in the timeliness of diagnosis by cancer stage; stage 3 and 4 cancers being diagnosed more quickly than stage 1 or 2 cancers. In addition, there is a great deal of dispersion irrespective of clinical stage with some 5–20% of patients requiring more than 6 months to get a diagnosis.

While guidelines exist regarding the evaluation of individuals with pulmonary nodules and lung cancer there are few guidance documents recommending timelines for lung nodule identification to diagnosis [6, 25, 26]. The NHS has recently published a handbook recommending that the time from nodule identification to diagnosis should be no more than 28 days [27]. Similar recommendations come from Canada and Norway [28, 29]In this study, approximately 75–80% of patients with stage 3 or 4 cancer were diagnosed within a 30-day period from nodule identification. The timeliness of diagnosis for those with stage 1 or 2 lung cancer was less with approximately 55–65% receiving a diagnosis within 30 days of nodule identification. These differences in timeliness of diagnosis by stage were statistically significant at the *p* < 0.001 level. This could be attributable to symptoms associated with advanced disease as well as the size and location of the more advanced tumors making biopsy access easier. In addition, differences in timeliness to lung cancer diagnosis between early stage and late stage tumors may have also been affected by a willingness on the part of the clinician or patient to “monitor” the lung nodule and obtain a repeat chest CT after a specific time interval following the index visit. Nonetheless, depending on tumor stage, some 25–45% of lung cancer patients exceeded these timing recommendations. The results from our study suggest that future guidelines on timeliness and quality of diagnosis should take into account clinical stage at presentation*.*

Among those patients with an SPN diagnosis but no evidence of lung cancer, follow-up procedures, the majority of which were referral visits or additional chest x-rays, were performed on the majority of this patient population. This contrasts with the findings of Pyenson et al. who studied administrative claims data (i.e., MarketScan) and reported that only 36% received follow-up care [30]. However, Pyenson and colleagues did not include chest imaging or E&M as part of their algorithm and these constituted 60–90% of follow-up procedures in our study. Ideally imaging should be conducted before the E&M but the data from this study highlight that this is not always the case. Coding and clinical care are not that similar and these administrative databses lack the granularity to address this issue. The use of PET, non-surgical biopsy and surgical resection as follow-up measures were quite similar between our study and theirs (3.4% vs 3.6, 2.9% vs 4.9, 0.4% vs 1% respectively).

The distribution of biopsies among lung cancer patients in our study mirrored those of others [31–34]. The pattern was slightly different for the SPN cohort in that approximately 70% of the biopsies were bronchoscopies compared to 48% in the lung cancer cohort. In addition, the biopsy rate was much lower in the SPN cohort relative to the lung cancer cohort (6% vs 79% respectively). Multiple biopsies were common in both groups occurring in 25% of the lung cancer group and in 35.5% of the SPN cohort. The frequency of multiple biopsies in the lung cancer cohort was lower in our study relative to others possibly attributable to the fact that we did not include biopsies performed after the diagnosis date (i.e., for staging purposes) [33–35]. Complications from biopsy procedures were rare in both patient cohorts with pneumothorax occurring in 4–5% of those undergoing CT guided biopsy attributable, in part, to the use of a conservative algorithm to insure an identified complication was the result of a specific procedure. (see Appendix 1 for codes).

Currently most lung cancer patients present with advanced disease. Our study is no different in that 69.2% of patients were diagnosed as stage 3 or 4. As screening is introduced more widely, the number of small nodules detected will increase, and most of these will be benign [36]. Guidelines that use a one-timeline for all can create counter-productive incentives, leading to unnecessary biopsies and ignore the value of careful observation and follow-up of low probability nodules. Guidelines on timeliness of care should take into account clinical stage at diagnosis and a more nuanced approach should be applied. Finally, technological improvements in the ability to access and biopsy small nodules in peripheral locations will facilitate more aggressive management of the SPN without compromising patient safety.

This study is not without limitations. This is a retrospective study that is associated with confounding factors for biopsies and treatment procedures. The patient population comes from two Louisiana healthcare systems and the patient pathways from nodule identification to diagnosis may not be generalizable to other healthcare systems throughout the country. However, findings regarding the stage at diagnosis are consistent with those previously reported throughout the literature. In addition, the linking of data from REACHnet with the Louisiana Tumor Registry resulted in many patients being excluded from the final cohort per protocol. For example, almost half of the patients with a diagnosis of lung cancer in REACHnet were excluded from the final sample because they had no record in the LTR or no record in the Tulane or Ochsner healthcare system. The impact of these patients on the patient pathway results is unknown.

## Conclusions

There are significant differences in the timeliness of diagnosis by cancer stage; stage 3 and 4 cancers being diagnosed more quickly than stage 1 or 2 cancers. In addition, there is a great deal of dispersion irrespective of clinical stage with some 5–20% of patients requiring more than 6 months to get a diagnosis. These results suggest that future guidelines on timeliness and quality of diagnosis should take into account clinical stage at presentation*.* More aggressive screening and referral coupled with timely diagnosis of earlier stage lung cancer and more aggressive follow-up of SPN will have the largest impact on patient outcomes.

## Supplementary Information

**Additional file 1: Appendix 1.** Master Code list.

**Additional file 2: Appendix 2.** Biopsy Complications by Lung Cancer and SPN Cohorts.

## Data Availability

The data that support the findings of this study are available from the corresponding author upon reasonable request.

## Abbreviations

LTR: Louisiana Tumor Registry
REACHnet: Research for Action Network
NCI: National Cancer Institute
SEER: Surveillance, Epidemiology, and End Results
GPID: Global Patient Identification
DCIFIRHD: Distributed Common Identity for the Integration of Regional Health Data
HashIDs: Hash Identifiers
ICD-9-CM: International Classification of Diseases, Ninth Revision, Clinical Modification
ICD-10-CM: International Classification of Diseases, Tenth Revision, Clinical Modification
ICD-O-3: International Classification of Diseases for Oncology, Third Revision
CPT: Current Procedural Terminology
SPN: Suspicious pulmonary nodule
LDCT: Low-Dose Computed Tomography
DCO: Death Certificate only
E&M: Evaluation and Monitoring
PET CT: Positron Emission Tomography-Computed Tomography
MRI: Magnetic Resonance Imaging
EHR: Electronic health record
SD: Standard deviation
