# Supplementary material for: Understanding the patient journey to diagnosis of lung cancer
Source: BMC Cancer. 2021 Apr 14;21:402. doi: 10.1186/s12885-021-08067-1 (PMC8045203; doi:10.1186/s12885-021-08067-1)
Supplement: Supplementary file 1 — Additional file 1: Appendix 1. Master Code list. [file 12885_2021_8067_MOESM1_ESM.docx]

**Title: Understanding the Patient Journey to Diagnosis of Lung Cancer**

**Authors:** Yichen Zhang^1^, Michael J. Simoff^2*^, David Ost^3^, Oliver J. Wagner^4^, James Lavin^5^, Beth Nauman^6^, Mei-Chin Hsieh^7^, Xiao-Cheng Wu^8^, Brian Pettiford^9^, Lizheng Shi^10^

**Author Affiliation:**

^1,10^ Tulane University, Department of Health Policy and Management, School of Public Health and Tropical Medicine. 1440 Canal Street, Suite 1900, New Orleans, LA 70112. [yzhang23@tulane.edu](mailto:yzhang23@tulane.edu); [lshi1@tulane.edu](mailto:lshi1@tulane.edu).

^2^ Pulmonary & Critical Care Medicine, Henry Ford Hospital, 2799 West Grand Boulevard, Detroit, MI 48202. [msimoff1@hfhs.org](mailto:msimoff1@hfhs.org).

^3^ University of Texas MD Anderson Cancer Center, Department of Pulmonary Medicine, Houston, TX 77030. [dost@mdanderson.org](mailto:dost@mdanderson.org).

^4,5^ Intuitive, 1020 Kifer Road, Sunnyvale, CA 94086. [oliver.wagner@intusurg.com](mailto:oliver.wagner@intusurg.com); [james.lavin@intusurg.com](mailto:james.lavin@intusurg.com).

^6^ Louisiana Public Health Institute, 1515 Poydras Street #1200, New Orleans, LA 70112. [bnauman@lphi.org](mailto:bnauman@lphi.org).

^7,8^ Louisiana State University Health Science Center, 433 Bolivar St, New Orleans, LA 70112. [mhsieh@lsuhsc.edu](mailto:mhsieh@lsuhsc.edu); [xwu@lsuhsc.edu](mailto:xwu@lsuhsc.edu).

^9^ Ochsner Health System, 1514 Jefferson Highway, Jefferson, LA 70121. [brian.pettiford@ochsner.org](mailto:brian.pettiford@ochsner.org).

^*^**Author for Correspondence:**

Michael J. Simoff, MD, FACP, FCCP

Director, Bronchoscopy and Interventional Pulmonology

Director, Lung Cancer Screening Program

Pulmonary and Critical Care Medicine

Henry Ford Hospital

Professor of Medicine, FTA

Wayne State University School of Medicine

2799 West Grand Boulevard

Detroit, MI 48202

Email: [msimoff1@hfhs.org](mailto:msimoff1@hfhs.org)

Telephone: 313-916-4406

Fax: 313-916-9102

**Appendix 1: Master Code list**

**All procedure codes listed below were mapped to ICD-9 and ICD-10 to capture IP procedures as appropriate**

**Suspicious Pulmonary Nodule**

| ICD-9 | 786.6, 793.1 |
| --- | --- |
| ICD-10 | R91.1, R91.8 |

**LDCT Screening**

| ICD-9 | V76.0 |
| --- | --- |
| ICD-10 | Z12.2 |
| HCPC | G0297, G0296, S8032 |

**Chest CT**

| CPT | 71250, 71260, 71270, 71010, 71020, 74176, 74178 |
| --- | --- |

**Evaluation & Management – must also have one of the lung cancer diagnosis codes listed below to qualify as a related visit**

| CPT | 99211-99213, 99251-99255, 99201-99205, 99212-99215 |
| --- | --- |

**Lung cancer diagnosis**

| ICD-9 Primary Malignancy | 162.0, 162.2, 162.3, 162.4, 162.5, 162.8, 162.9 |
| --- | --- |
| ICD-10 Primary Malignancy | C33, C34.00, C34.01, C34.02, C34.10, C34.11, C34.12, C34.2, C34.30, C34.31, C34.32, C34.80, C34.81, C34.82, C34.90, C34.91, C34.92 |
| ICD-9 Secondary Malignancy | 197.0 |
| ICD-10 Secondary Malignancy | C78.00, C78.01, C78.02, C78.1, C78.2, C78.3, C78.30, C78.39 |
| ICD-9 Benign Malignancy | 212.2, 212.3, 212.8, 212.9 |
| ICD-10 Benign Malignancy | D14.2, D14.3, D14.30, D14.31, D14.32, D14.4 |
| ICD-9 Pathologist uncertain behavior | 235.7, 235.9, 239.1 |
| ICD-10 Pathologist uncertain behavior | D38.0, D38.1, D38.5, D38.6, D49.1 |

**EBUS & Mediastinoscopy: all procedures cross walked to ICD-9 and ICD-10**

| CPT | 31652, 31653, 31654, 31620, 31899, 39000, 39010, 39400, 39401, 39402, 32606, 32662, 38746, 39220 |
| --- | --- |

**Biopsy Procedure:**

| Percutaneous CPT | 32405 |
| --- | --- |
| Surgical | 32096, 32097, 32484, 32505,32507, 32607, 32608, 32609, 32668 |
| Bronchoscopy | 31627, 31622, 31623, 31624, 31625, 31629, 31633, 31628, 31632, 31640, 31635, 31636, 31637, 31638, 31641, 31643, 31645, 31646 |

**PET Scan**

| CPT | 78811, 78814, 78812, 78813, 78815, 78816 |
| --- | --- |

**Brain & Bone Scan**

| Brain Scan CPT | 70552, 70551, 70553 |
| --- | --- |
| Bone Scan CPT | 78306, 78315, 78300, 78305 |

| Supplement: ICD-9/ICD-10 and CPT Codes used to Identified Post-Procedural Complication and Treatment (where applicable) | | | | | | |
| --- | --- | --- | --- | --- | --- | --- |
| **Complication types** | **ICD9CM** | **ICD10CM** | **CPT** | **ICD9 Procedure** | **ICD10 Procedure** |  |
| 1. Pneumothorax - Day 1 | 512.1 | J95.811 |  |  |  |  |
|  |  |  |  |  |  |  |
| 2. Pneumothorax requiring a chest tube – Day 1 | 512.1 | J95.811 | 32554 | 34.04 | 0W9930Z |  |
|  |  |  | 32555 | 34.06 | 0W9B30Z |  |
|  |  |  | 32551 | 34.09 | 0W9940Z |  |
|  |  |  |  | 34.01 | 0W9B40Z |  |
|  |  |  |  |  | 0BHQ3YZ |  |
|  |  |  |  |  | 0WP830Z |  |
| 3. Hemorrhage – Day 1 | 459.0 | R58 |  |  |  |  |
|  | 998.11 | J95.830 |  |  |  |  |
|  | 998.2 | J95.831 |  |  |  |  |
|  | 998.12 | J95.71 |  |  |  |  |
|  |  | J95.62 |  |  |  |  |
|  |  | R04.9 |  |  |  |  |
| 4. Prolonged Air Leak - >Day 5 | 512.2 | J93.0 |  |  |  |  |
|  |  | J93.82 |  |  |  |  |
|  |  | J95.812 |  |  |  |  |

| **Biopsy Procedure** | **CPT Codes** | **Complications to query** |
| --- | --- | --- |
| CT Guided Biopsy | 32405 | 1-4 |
| Bronchoscopy including EBUS | 31629, 31620, 31652, 31653, 31633,31628, 31627, 31654, 31632, 31623, 31622, 31624, 31625, 31640, 31635, 31636, 31637, 31638, 31641, 31643, 31645, 31646 | 1-4 |
| Surgical Biopsy* including mediastinoscopy, and biopsy of the pleura (as a complication only) | 32096, 32097, 32484, 32507, 32607, 32608, 32609, 32400, 32554, 32555, 39400, 39401, 39402, 39000, 39010, 38746, 39220, 32662, 32605, 32606 | 3-4 |

*CPT 32505, 32668 are both diagnostic and therapeutic and are not included as part of the complications analysis. CPT 31899 is a miscellaneous code cannot be used in a complications analysis

**CT Guided Biopsy**

| 32405 | Biopsy, lung or mediastinum, percutaneous needle |
| --- | --- |

**Bronchoscopy**

| 31627 | Bronchoscopy, rigid or flexible, including fluoroscopic guidance, when performed; with computer-assisted, image-guided navigation (List separately in addition to code for primary procedure[s]) |
| --- | --- |
| 31622 | Bronchoscopy, rigid or flexible, including fluoroscopic guidance, when performed; diagnostic, with cell washing, when performed (separate procedure) |
| 31623 | Bronchoscopy, rigid or flexible, including fluoroscopic guidance, when performed; with brushing or protected brushings |
| 31624 | Bronchoscopy, rigid or flexible, including fluoroscopic guidance, when performed; with bronchial alveolar lavage |
| 31625 | Bronchoscopy, rigid or flexible, including fluoroscopic guidance, when performed; with bronchial or endobronchial biopsy(s), single or multiple sites |
| 31629 | "Bronchoscopy, rigid or flexible, including fluoroscopic guidance, when performed; with transbronchial needle aspiration biopsy(s), trachea, main stem and/or lobar bronchus(i) |
| 31633 | " |
| 31628 | Bronchoscopy, rigid or flexible, including fluoroscopic guidance, when performed; with transbronchial needle aspiration biopsy(s), each additional lobe (List separately in addition to code for primary procedure) ADD ON To 31629 |
| 31632 | Bronchoscopy, rigid or flexible, including fluoroscopic guidance, when performed; with trans bronchial lung biopsy(s), single lobe |
| 31640 | Bronchoscopy, rigid or flexible, including fluoroscopic guidance, when performed; with transbronchial lung biopsy(s), each additional lobe (List separately in addition to code for primary procedure) ADD ON TO 31628 |
| 31635 | Bronchoscopy, rigid or flexible, including fluoroscopic guidance, when performed, with excision of tumor |
| 31636 | Bronchoscopy, rigid or flexible, including fluoroscopic guidance, when performed, with removal of foreign body -( was used pre 2016 for removal of a stent) |
| 31637 | Bronchoscopy, rigid or flexible, including fluoroscopic guidance, when performed, with placement of stents |
| 31638 | Bronchoscopy, rigid or flexible, including fluoroscopic guidance, when performed, each additional stent |
| 31641 | Bronchoscopy, rigid or flexible, including fluoroscopic guidance, when performed, with stent revision |
| 31643 | Bronchoscopy, rigid or flexible, including fluoroscopic guidance, when performed, with destruction of tumor |
| 31645 | Bronchoscopy, rigid or flexible, including fluoroscopic guidance, when performed, with placement of catheters |
| 31646 | Bronchoscopy, rigid or flexible, including fluoroscopic guidance, when performed, with therapeutic aspiration |

**Surgery**

| 32607 | Thoracoscopy; with diagnostic biopsy(ies) of lung infiltrate(s) (eg, wedge, incisional), unilateral |
| --- | --- |
| 32602 | Thoracoscopy, diagnostic; with diagnostic biopsy(ies) of lung nodule(s) or mass(es) (eg, wedge, incisional), unilateral (code deleted and replaced with 32608) |
| 32608 | Thoracoscopy, diagnostic; with diagnostic biopsy(ies) of lung nodule(s) or mass(es) (eg, wedge, incisional), unilateral |
| 32609 | Thoracoscopy, surgical with biopsy(ies) of pleura |
| 32096 | (this code series replaces 32095 from Wiener) Thoracotomy, with diagnostic biopsy(ies) of lung infiltrate(s) (eg, wedge, incisional), unilateral |
| 32097 | Thoracotomy, with diagnostic biopsy(ies) of lung nodule(s) or mass(es) (eg, wedge, incisional), unilateral |
| 32507 | add on code: with diagnostic wedge resection followed by anatomic lung resection. +32507 must be reported with 32440, 32442, 32445, 32480, 32482, 32486, 32488, 32503, 32504 |
| 32484 | Removal of lung, other than pneumonectomy; single segment (segmentectomy) |
| 32607 | Thoracoscopy; with diagnostic biopsy(ies) of lung infiltrate(s) (eg, wedge, incisional), unilateral |
| 32602 | Thoracoscopy, diagnostic; with diagnostic biopsy(ies) of lung nodule(s) or mass(es) (eg, wedge, incisional), unilateral (code deleted and replaced with 32608) |
| 32608 | Thoracoscopy, diagnostic; with diagnostic biopsy(ies) of lung nodule(s) or mass(es) (eg, wedge, incisional), unilateral |
| 32609 | Thoracoscopy, surgical with biopsy(ies) of pleura |
| 32554 | Thoracentesis, needle or catheter, aspiration of the pleural space; without imaging guidance |
| 32555 | Thoracentesis, needle or catheter, aspiration of the pleural space; with imaging guidance |
| 32400 | Biopsy, pleura, percutaneous needle |
| 39000 | Mediastinotomy |
| 39010 | Mediastinotomy, transthoracic approach |
| 39400 | Mediastinoscopy, includes biopsy(ies), when performed |
| 39401 | Mediastinoscopy; includes biopsy(ies) of mediastinal mass (eg, lymphoma), when performed |
| 39402 | Mediastinoscopy; with lymph node biopsy(ies) (eg, lung cancer staging) |
| 32605 | Thorascopy, mediastinal space, without biopsy |
| 32606 | Thorascopy, mediastinal space, with biopsy |
| 32662 | Thorascopy, surgical with excision of mediastinal cyst, tumor or mass |
| 38746 | Thoracic lymphadenectomy |
| 39220 | Resection of a mediastinal tumor |

| **Appendix 2: Biopsy Complications by lung Cancer and SPN cohorts** | | |
| --- | --- | --- |
|  | **Lung Cancer N=1496** | **SPN, no Cancer N=15,978** |
|  | 1179/1496 patients had a biopsy procedure (78.81%) | 932/15978 patients had a biopsy procedure (5.83%) |
| **Total Patients with complications** | 38 patients had a complication (3.22%) | 25 patients had a complication (2.68%) |
| **CT Guided Biopsy only N (%)** | 628 procedures | 271 procedures |
| Day one: |  |  |
| pneumothorax | 27 (4.30) | 14 (5.17) |
| pneumothorax requiring chest tube | 2 (0.32) | 0 (0.00) |
| hemorrhage | 0 (0.00) | 2 (0.74) |
| >5 Days: |  |  |
| air leak | 1 (0.16) | 1 (0.37) |
|  |  |  |
| **Bronchoscopy, including EBUS** | 710 procedures | 970 procedures |
| Day one: |  |  |
| pneumothorax | 6 (0.85) | 2 (0.21) |
| pneumothorax requiring chest tube | 2 (0.28) | 0 (0.00) |
| hemorrhage | 2 (0.28) | 1 (0.10) |
| >5 Days: |  |  |
| air leak | 2 (0.28) | 4 (0.41) |
|  |  |  |
| **Surgical biopsy, including mediastinoscopy and pleural biopsy*** | 198 procedures | 211 procedures |
| Day one: |  |  |
| hemorrhage | 1 (0.51) | 0 (0.00) |
| >5 Days: |  |  |
| air leak | 3 (1.52) | 3 (1.42) |
| *pleural biopsy included in surgical totals: | 68 | 49 |
